# Combining active restoration and targeted grazing to establish native plants and reduce fuel loads in invaded ecosystems

**DOI:** 10.1002/ece3.4642

**Published:** 2018-12-11

**Authors:** Lauren M. Porensky, Barry L. Perryman, Matthew A. Williamson, Matthew D. Madsen, Elizabeth A. Leger

**Affiliations:** ^1^ Rangeland Resources and Systems Research Unit USDA Agricultural Research Service Fort Collins Colorado; ^2^ Deparment of Agriculture, Nutrition and Veterinary Sciences University of Nevada Reno Nevada; ^3^ Department of Environmental Science and Policy University of California Davis California; ^4^ Department of Plant and Wildlife Sciences Brigham Young University Provo Utah; ^5^ Department of Natural Resources and Environmental Science University of Nevada Reno Nevada

**Keywords:** *Bromus tectorum*, brownstrip, forage kochia, greenstrip, precision restoration, surfactant

## Abstract

Many drylands have been converted from perennial‐dominated ecosystems to invaded, annual‐dominated, fire‐prone systems. Innovative approaches are needed to disrupt fire‐invasion feedbacks. Targeted grazing can reduce invasive plant abundance and associated flammable fuels, and fuelbreaks can limit fire spread. Restored strips of native plants (native greenstrips) can function as fuelbreaks while also providing forage and habitat benefits. However, methods for establishing native greenstrips in invaded drylands are poorly developed. Moreover, if fuels reduction and greenstrip establishment are to proceed simultaneously, it is critical to understand how targeted grazing interacts with plant establishment. We determined how targeted grazing treatments interacted with seed rate, spatial planting arrangement (mixtures vs. monoculture strips), seed coating technology, and species identity (five native grasses) to affect standing biomass and seeded plant density in experimental greenstrips. We monitored for two growing seasons to document effects during the seedling establishment phase. Across planting treatments, ungrazed paddocks had the highest second‐year seeded plant densities and the highest standing biomass. Paddocks grazed in fall of the second growing season had fewer seedlings than paddocks grazed in spring, five months later. High seed rates minimized negative effects of grazing on plant establishment. Among seeded species, *Elymus trachycaulus* and *Poa secunda *had the highest second‐year densities, but achieved this via different pathways. *Elymus trachycaulus* produced the most first‐year seedlings, but declined in response to grazing, whereas *P. secunda* had moderate first‐year establishment but high survival across grazing treatments. We identified clear tradeoffs between reducing fuel loads and establishing native plants in invaded sagebrush steppe; similar tradeoffs may exist in other invaded drylands. In our system, tradeoffs were minimized by boosting seed rates, using grazing‐tolerant species, and delaying grazing. In invaded ecosystems, combining targeted grazing with high‐input restoration may create opportunities to limit wildfire risk while also shifting vegetation toward more desirable species.

## 1 INTRODUCTION

Fire size, frequency and severity are increasing due to climate change and land use change, and shifting fire regimes are altering ecosystem function and ecosystem service provisioning globally (Abatzoglou, Kolden, Williams, Lutz, & Smith, 2017; Dennison, Brewer, Arnold, & Moritz, 2014; Moritz et al., 2014). With a warming climate, the most effective strategies to manage increased fire activity will be those resulting in self‐sustaining plant communities able to resist or recover from fire without the need for continuing management inputs (Suding et al., 2015). Nevertheless, many current approaches to wildland fire mitigation rely on the repeated use of brush control, herbicide‐ or mechanically generated fuelbreaks (“brownstrips”), or ongoing active fire suppression (Wildland Fire Executive Council, 2014). Strategies that rely on restoring historic vegetation structure, such as recent efforts in forested systems (Fule, Covington, & Moore, 1997), can provide lasting benefits by increasing long‐term resilience and resistance to future fire (Hanberry, Noss, Safford, Allison, & Dey, 2015).

In many dryland shrublands, wildfires have been historically small and infrequent because fires rarely spread through sparse, discontinuous vegetation (Klinger & Brooks, 2017). However, historic plant community composition and structure in many of these ecosystems have been drastically altered by the introduction of invasive annual grasses (e.g., *Bromus tectorum *L., *Bromus rubens *L., and *Schismus* spp. P. Beauv.) that increase fuel loads and fuel continuity as biomass accumulates between shrubs and perennial grasses, ultimately increasing the likelihood of fire ignition and spread (Balch, Bradley, D'Antonio, & Gómez‐Dans, 2013; Brooks et al., 2004; Davies & Nafus, 2013). Feedbacks between fire and invasive species can cause widespread ecosystem conversions, for example from shrublands to grasslands dominated by nonnative annuals (Alba, Skalova, McGregor, D'Antonio, & Pysek, 2015; Balch et al., 2013; D'Antonio & Vitousek, 1992). As a result, efforts to restore invaded shrublands are increasingly focused on reducing fuel loads or fuel connectivity and restoring fire‐resistant vegetation structure (Gray & Dickson, 2015, 2016; Pellant, 1989). To provide long‐term fire resistance, restoration should include replacing fire‐prone invasive species with less flammable species, but this has proved elusive in areas with high fire frequency and intense competition from invasive species (Duniway, Palmquist, & Miller, 2015; Eiswerth & Shonkwiler, 2006; Pyke, Wirth, & Beyers, 2013).

**Figure 1:**
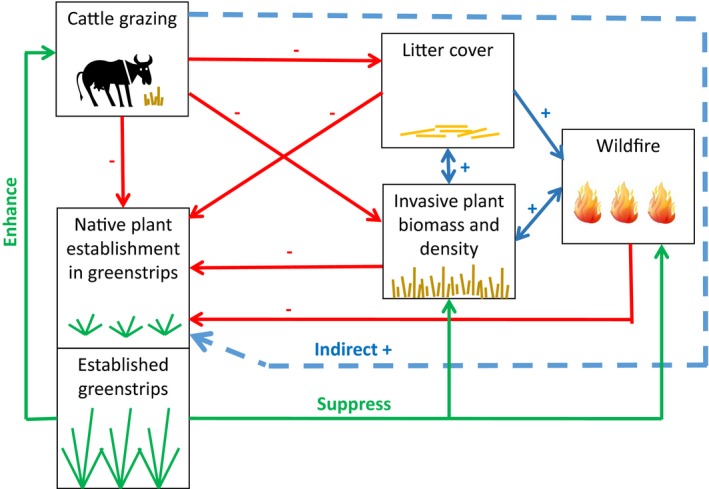
Hypothesized direct and indirect effects of grazing on native plant establishment in greenstrips (red and blue arrows), and hypothesized effects of established greenstrips on invasion, wildfire and season‐long forage availability (green arrows). Second‐year results provide evidence for negative direct effects of cattle grazing on both seedling establishment and invasive plant biomass, but no evidence for cattle grazing effects on litter cover or invasive plant density

In invaded drylands, developing management strategies that can simultaneously increase the establishment of fire‐resistant plant species and decrease the risk of fire spread would be an important step toward restoring fire regimes characterized by small, infrequent wildfires. Many dryland ecosystems are currently used for livestock grazing, making targeted grazing an attractive option for vegetation management. Targeted grazing is designed to achieve specific vegetation management goals, such as reduced fuel loads or reduced cover of invasive plants, via specified timing, duration, and intensity of use (Frost & Launchbaugh, 2003). Targeted grazing can disrupt invasion‐fire feedback cycles by reducing fuel loads and connectivity (Davies, Bates, Boyd, & Svejcar, 2016; Davies, Bates, Svejcar, & Boyd, 2010; Davies, Boyd, Bates, & Hulet, 2016; Davies, Gearhart, Boyd, & Bates, 2017; Davies, Svejcar, & Bates, 2009; Diamond, Call, & Devoe, 2009; Schmelzer et al., 2014). By reducing litter that facilitates annual grass dominance (Beckstead & Augspurger, 2004; Jones, Chambers, Board, Johnson, & Blank, 2015), targeted fall grazing can also negatively impact annual grass performance in subsequent years with minimal negative effects on perennial bunchgrasses (Schmelzer et al., 2014; Trowbridge et al., 2013). Further, targeted spring grazing can reduce annual grass seed production (Diamond, Call, & Devoe, 2012). Via these mechanisms, targeted grazing should reduce competition between invasive annuals and restored, fire‐resistant plants. However, it is unclear whether the net effects of targeted grazing practices on planted seedlings are positive or negative (Figure 1). Defoliation and uprooting are likely to reduce the survival and growth of individual seedlings, but targeted grazing treatments may indirectly assist planted seedlings by reducing litter, biomass, and densities of competitive invasive annuals, and by reducing wildfire risk (Figure 1). Targeted grazing can reduce native grass seedbanks (Diamond et al., 2012; Schmelzer, 2009), but to our knowledge, no studies have investigated impacts of targeted grazing on native seedling establishment in western United States drylands.

Like targeted grazing, fuelbreaks have been used as a means of reducing fuel loads and fuel connectivity, particularly in areas where roads provide a potential ignition source or to protect infrastructure in wildland‐urban interfaces (Pellant, 1989). Without continued maintenance, mechanically or chemically created fuelbreaks (“brownstrips”) can exacerbate invasive species challenges (Merriam, Keeley, & Beyers, 2006). Greenstrips, which are linear plantings designed to reduce fire size or frequency and prevent fire spread into uninvaded or restored areas, are an alternative approach aimed at creating patches of self‐sustaining, fire‐resistant vegetation (Pellant, 1989). To resist wildfire, greenstrips must either include vegetation with low biomass and large gaps or vegetation that maintains high moisture content during the fire season (Monaco, Waldron, Newhall, & Horton, 2003; Robbins, Staub, & Bushman, 2016). Greenstrips in the western US have often relied on nonnative plant species (Harrison et al., 2002), but greenstrips composed of native plants have the potential to provide added benefits such as native biodiversity and wildlife habitat variety (Hulvey et al., 2017) while avoiding the unintended spread of introduced species (Gray & Muir, 2013). Further, relative to more diffuse native plant restoration approaches, native greenstrips may lead to greater return‐on‐investment because a greenstrip approach allows practitioners to concentrate effort and resources into small, spatially strategic locations (Hulvey et al., 2017).

It remains unclear how to best produce native greenstrips, or how to combine greenstrips with targeted grazing to disrupt fire‐invasion feedbacks. Establishing native plants from seed in highly invaded settings remains challenging (Eiswerth & Shonkwiler, 2006). Existing work on restoration in drylands suggests that seedling establishment is a critical bottleneck in these ecosystems (James, Svejcar, & Rinella, 2011), with survivorship increasing substantially after the first or second growing season (Leger & Goergen, 2017). Our work explored the separate and combined efficacy of five approaches that could potentially be utilized to alter competitive dynamics and increase seedling establishment in dryland, fire‐prone restoration settings: using seed coatings to increase water availability (Madsen, Kostka, Inouye, & Zvirzdin, 2012), choosing species that can compete at the seedling stage with invasive annuals (Rowe & Leger, 2011), bolstering seed rates (Mazzola et al., 2010), using spatial separation to reduce competition among planted species (Porensky, Vaughn, & Young, 2012), and using targeted spring and fall grazing to reduce invasive plant competition and associated wildfire risk (Davies et al., 2017; Schmelzer et al., 2014).

We implemented a 164‐ha experiment at a highly invaded site in the Great Basin region of the western US. In this region, almost 1/3 of the land area (210,000 km^2^) is highly invaded by annual species that burn with greater frequency than vegetation in uninvaded areas (Bradley et al., 2018). The invasion of *B. tectorum* in this region has caused a widespread loss of wildlife habitat, livestock forage, soil health, plant genetic diversity, and other ecosystem services (DiTomaso, 2000; Eiswerth, Darden, Johnson, Agapoff, & Harris, 2005; Mack, 1981). *Bromus tectorum* increases wildfire frequency and extent (Balch et al., 2013; Davies & Nafus, 2013), making this an excellent system for investigating the efficacy of targeted grazing and native greenstrip plantings as tools for disrupting wildfire‐invasion feedbacks. We hypothesized that:

1. Targeted fall and spring grazing during the second growing season would reduce invasive species biomass, litter cover and invasive plant densities via direct consumption of plants and seeds and trampling of litter.
2. Targeted grazing during the second growing season would reduce densities of seeded species in experimental greenstrips, and, because plants are actively growing, spring grazing would have stronger negative effects than fall grazing.
3. Higher seed rates, seed coatings, use of competitive native grasses, and spatially segregated planting arrangements would enhance seedling establishment, mitigating any negative effects of targeted grazing.

## 2 METHODS

### 2.1 Study site

Our site was in northern Nevada at the TS Ranch (Elko Land and Livestock Company; 40.843–40.895 N, 116.509–116.554 W). The site is a southeast‐facing, gently sloping alluvial fan on loamy soils, and the climate is typical of a cold desert (250 mm annual precipitation, average daily temperature is −3°C in January and 22°C in July, PRISM Climate Group, [Link]). Precipitation was average during the establishment year (248 mm from 10/1/2014–9/30/2015) and above average during the second season (323 mm from 10/1/2015–9/30/2016; Supporting Information Table S1). Vegetation was dominated by *B. tectorum* and nonnative annual forbs including *Sisymbrium altissimum *L., *Lepidium perfoliatum *L.*, Salsola tragus *L., *Ceratocephala testiculata *(Crantz) Roth, and *Chorispora tenella *(Pall.) DC. A few native species were present including *Poa secunda *J. Presl, *Elymus elymoides *(Raf.) Swezey, *Vulpia microstachys *(Nutt.) Munro, *Microsteris gracilis *(Hook.) Greene*, Crepis occidentalis *Nutt., *Amsinckia tessellata *A. Gray*, Lappula occidentalis *(S. Watson) Greene, and *Mentzelia albicaulis *(Hook.) Torr. & A. Gray. Based on vegetation composition of surrounding unburned areas with similar land position, we hypothesize that this site had vegetation typical of lowland sagebrush steppe (dominated by *Artemisia tridentata *Nutt. ssp.* wyomingensis *Beetle & Young, perennial bunchgrasses, and annual forbs) prior to invasion.

### 2.2 Experimental design

**Figure 2:**
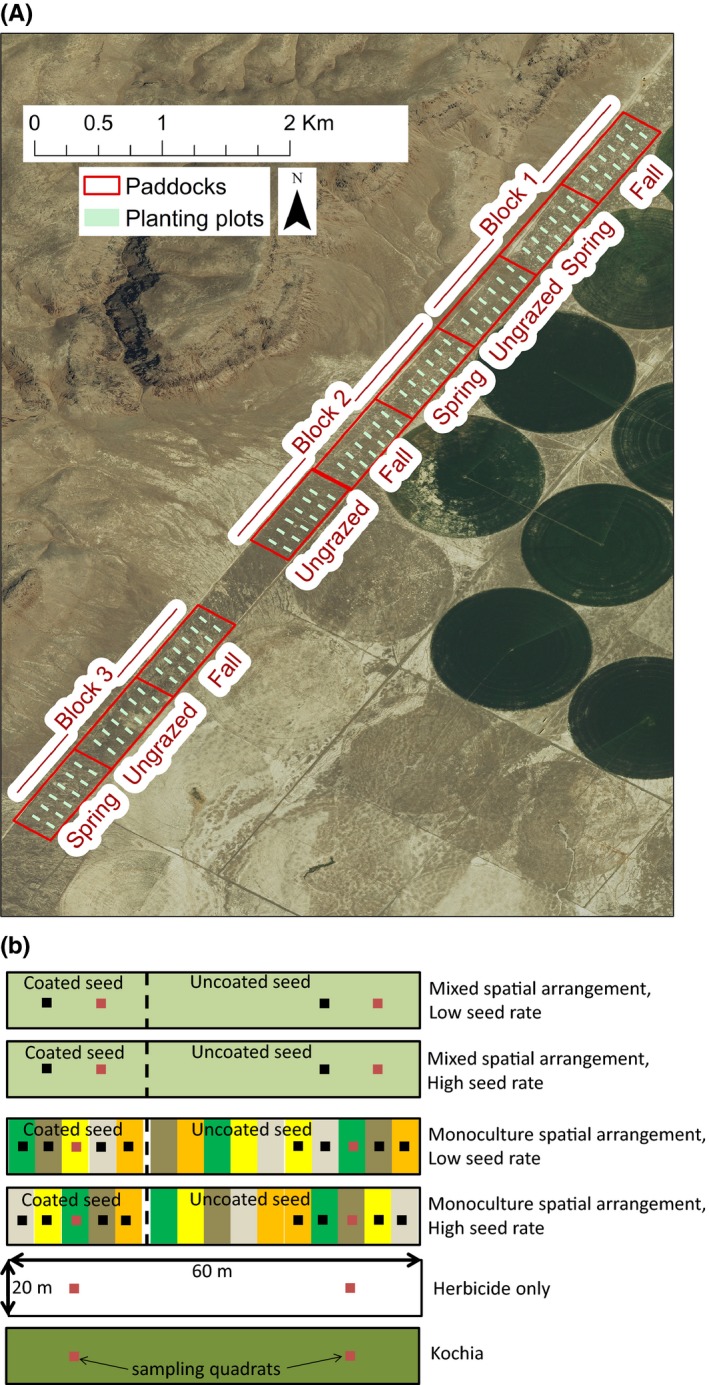
The experiment had a hierarchical design with three levels. (a) Blocks and paddock‐scale treatments. Each of three blocks contained three 18.21 ha paddocks randomly assigned to different grazing treatments. Each paddock contained twelve 0.12 ha planted plots and twelve unseeded control plots. Unseeded control plots were randomly interspersed among planted plots (>15 m and <50 m from any planted plot). (b) Plot‐ and subplot‐scale treatments. The twelve plots within each paddock were randomly assigned to six different treatments. Plots planted with grasses also included three 20 × 20 m subplots, one of which was randomly assigned to the coated seed treatment. In monoculture treatments, colors represent different species. Plant cover and seeded species densities were sampled in both red and black quadrats; nonseeded species densities were only sampled in red quadrats

The experimental design was hierarchical with three levels (split‐split plot, Smith, Pullan, & Shiel, 1996). At the broadest scale, a randomized complete block design was employed for grazing treatments. Nine 18.21 ha paddocks were arranged into three blocks (Figure 2a). One paddock per block was randomly assigned to each of three grazing treatments: fall, spring, or no grazing. Blocks were arranged to capture potential variation associated with topographic and agricultural features (Figure 2a).

Within each paddock (middle level of the hierarchical design), we established twelve 0.12 ha experimental greenstrip plots (20 × 60 m) separated by ≥50 m. Eight of these plots were randomly assigned to four native grass restoration treatments, with two replicates of each treatment per paddock. Treatments included all combinations of two spatial arrangement treatments and two seed rate treatments (Figure 2b). Spatial arrangement treatments included “monoculture strip” plots, in which species were seeded separately into adjacent 4‐m wide strips, and “mixture” plots, in which the same amount of seed was mixed across all species before planting. Seed rate treatments included 1× or 2× seed rates, where 1× rates followed rangeland restoration guidelines for each species based on a total pure live seed (PLS) rate of 646 seeds/m^2^.

The seed mix included five grass species with a diversity of life history strategies, which were selected from a pool of species native to the region. Species included two grazing‐tolerant, early‐season perennial bunchgrasses (*E. elymoides*, squirreltail, and *P. secunda*, Sandberg bluegrass) and one annual grass (*Vulpia microstachys *(Nutt.) Munro, small fescue). We expected these species to compete well with *B. tectorum* due to similar phenology (Leger, Goergen, & Forbis de Queiroz, 2014). Two taller, deeper‐rooted perennial grasses that typically stay green into the fire season were also included: one rhizomatous species (*Elymus trachycaulus* (Link) Gould ex Shinners, slender wheatgrass) and one bunchgrass (*Poa fendleriana *(Steud.) Vasey, muttongrass). Seed rates for the 1× treatment were as follows: *E. elymoides* 3.05 PLS kg/ha; *E. trachycaulus* 4.34 PLS kg/ha; *P. fendleriana* 0.65 PLS kg/ha; *P. secunda* 0.56 PLS kg/ha; and *V. microstachys* 0.61 PLS kg/ha.

At the finest scale of the experimental design (split‐split plot), each experimental greenstrip plot included three 20 × 20 m subplots, one of which was randomly selected for a coated seed treatment. Seeds of each species were coated with a nonionic alkyl terminated block copolymer surfactant coating based on C1–C4 alkyl ethers of methyl oxirane–oxirane copolymers (Aquatrols Corporation of America, Paulsboro, NJ). This surfactant has been used to increase the wettability of water‐repellent soils (Fernelius et al., 2017; Kostka, 2000) but also improves plant drought tolerance in wettable soils by reducing the time it takes for a root to rehydrate and by decreasing plant transpiration rates (Ahmed et al., 2018). Increased drought tolerance provided by the seed coating could produce more vigorous plants with greater seedling survival. Seed rates were determined prior to coating. In each paddock, we also included two 0.12 ha nonnative greenstrip plots seeded with forage kochia (*Bassia prostrata *(L.) A.J. Scott, 6.73 PLS kg/ha), and two 0.12 ha brownstrip plots sprayed with the targeted herbicide imazapic. Both of these techniques are commonly used to create firebreaks on *B. tectorum*‐invaded sites, and their inclusion allowed for direct comparisons with native grass greenstrip treatments.

All plots were initially sprayed with glyphosate in April 2014, prior to seeding (840 g/ha). Herbicide plots were resprayed in spring 2015 and 2016 with imazapic (420 g/ha). Plots assigned to seeding treatments were not resprayed and were seeded with a rangeland drill in October and November 2014. Grazing treatments were initiated in Fall 2015, providing a full growing season of deferment. Fall‐grazed paddocks were grazed by 25 cows (25 animal units [AUs]) for 7–9 days each between 15 October and 9 November 2015. Spring‐grazed paddocks were grazed by 29 yearlings (22 AUs) for 9–11 days each between 5 April and 4 May 2016. Grazing periods and grazing animals were determined based on availability of livestock, with animals split between paddocks for the time available and leaving not less than 112 kg/ha of standing crop.

### 2.3 Data collection

To address Hypothesis 1, we quantified standing biomass in June 2015, prior to grazing treatments, in each paddock by harvesting aboveground biomass rooted inside five 1‐m^2^ plots per paddock (four unseeded controls and one randomly selected native grass plot). Unseeded control plots were located randomly within each paddock but outside of planted plots (>15 m and <50 m from any planted plot). In November 2015 and May 2016, we applied the same method at 16 locations per paddock (four unseeded controls and all 12 treatment plots), and separated clipped biomass into four functional groups: *B. tectorum*, forbs, native grasses, and seedlings of planted species. Clip locations were shifted by 3–5 m between sampling periods to avoid resampling previously clipped areas.

To address Hypotheses 2 and 3, seedling emergence and aerial cover were sampled in May 2015 and May‐June 2016 at multiple locations within each plot (see Figure 2b for sampling locations). In 2016, we also sampled emergence and cover at 12 unseeded control plots per paddock. Unseeded controls were thus monitored within each grazing treatment. In 2015, we counted seedlings within 25 × 50 cm quadrat at each sampling location. In 2016, we sampled at the same locations but used a 50x50 cm quadrat to ensure that seeded species were encountered during density counts, due to density reductions between 2015 and 2016. At the same sampling locations, in each year, we visually estimated percent plant foliar cover (plant material that would intercept a raindrop) by species for both seeded and nonseeded species in a 1‐m^2^ quadrat. For portions of the quadrat without plant cover, we estimated cover of litter (detached plant tissue) and bare ground. Integer increments from 0%–100% were used for visual cover estimates. Because of small seedling sizes, it was difficult to confidently distinguish among some planted grass species (e.g, *Elymus elymoides* and *E. trachycaulus*) in mixture plots, so seeded species densities and aerial cover of seeded species were recorded in aggregate for these plots. Monoculture plots were used to evaluate differences in establishment success among seeded species. At two sampling stations per plot (Figure 2b), we also recorded densities of nonseeded species in a 25 × 25 cm quadrat.

### 2.4 Data analysis

To address Hypothesis 1, data on aboveground biomass, litter cover, and invasive species density and cover were analyzed for each year using linear mixed models. Random effects included block, paddock nested within block, and (for cover and density analyses only) plot nested within paddock and block. Fixed effects included grazing treatments, plot types (control and grass for biomass in June 2015; control, grass, herbicide and kochia for all other analyses), and their interaction. Though plots had not yet been grazed in June 2015, we included the planned grazing treatment in these models to test for pretreatment differences. These analyses were conducted with JMP (JMP®, Version 12. SAS Institute Inc., Cary, NC, 1989–2007). Data were transformed and variance‐weighted when necessary to meet model assumptions.

To address Hypotheses 2 and 3, seedling data were analyzed using generalized linear mixed models with a negative binomial distribution due to zero‐inflated count data. This distribution fit the data significantly better than several other potential distributions (e.g., Gaussian, Poisson, Gamma). We analyzed each year separately to better understand the factors influencing plant success at different demographic stages. For each year, we ran three models using seedlings of planted species per m^2^ as the response variable. For all models, random effects included block, paddock nested within block, and plot nested within paddock and block to account for the experiment's hierarchical design. Two quadrats located 4 m apart in the same plot had extremely high numbers of resident (nonseeded) *V. microstachys* and were therefore excluded from all analyses.

The first model (“grazing × establishment model”) determined if grazing treatments affected seedling densities and if plots planted with native grasses had different seedling densities than forage kochia plots, herbicide plots, or unplanted controls. For year one data, collected prior to initiation of grazing treatments, the only fixed effect in the model was plot type (grass or forage kochia; seedlings were not censused in control plots in year one). For second‐year data, fixed effects included plot type (grass, forage kochia, or control), grazing treatment, and their interaction. Herbicide plots were excluded from analysis of second‐year data because no seedlings were observed.

The second model (“planting strategies model”) assessed the effects of multiple planting methods on seedling densities, using only data from plots seeded with native grasses. Fixed effects included seed rate, spatial arrangement, seed coating, grazing treatment (for second‐year data), and all interactions among these factors. Seed coating subplot was added as an additional random effect nested within plot, paddock and block.

The third model (“grass species model”) assessed how species‐level differences affected seedling densities, using only data from monoculture grass plots. Fixed effects included species, seed rate, grazing treatment (for second‐year data), seed coating, and two‐ or three‐way interactions. Seed coating subplot was again included as a random effect. Seedling analyses were performed with the lme4 statistical package (Bates, Maechler, Bolker, & Walker, 2015) in R (version 3.3.1). Denominator degrees of freedom were determined conservatively based on the hierarchical experimental design (Pinheiro & Bates, 2000) and used to calculate *p*‐values. Results were considered significant at *p* < 0.05 and are reported as mean ± standard error (*SE*).

## 3 RESULTS

### 3.1 Grazing reduced standing biomass, but not litter or invasive plant densities

**Figure 3:**
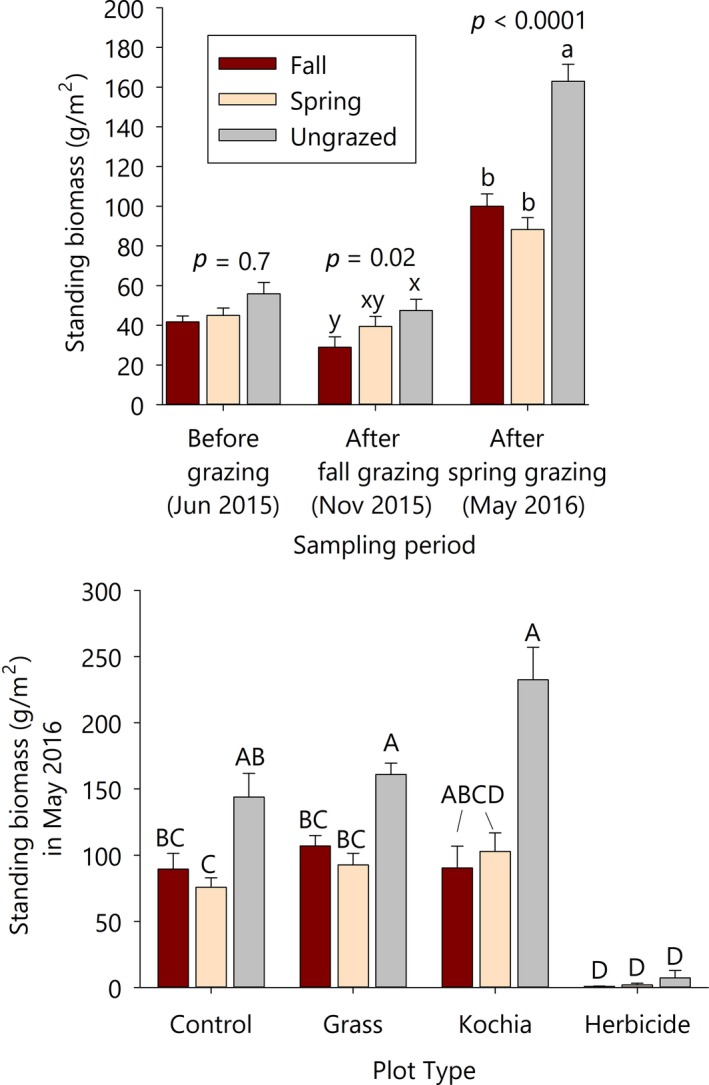
Effects of grazing treatments on standing biomass (a) across sampling dates (*p*‐values represent the main effect of grazing for each date) and (b) across plot types in May 2016 (grazing × type *p* < 0.0001). Control, or unseeded, plots were monitored within each paddock (across all three grazing treatments). June 2015 was prior to grazing, November 2015 was after fall grazing, and May 2016 was after spring grazing. Within a sampling date, treatments sharing letters do not differ significantly (Tukey HSD). Different capitalizations and letter groups are used to differentiate among results from different models.

Standing biomass did not differ among grazing treatments or between unseeded controls and seeded plots before grazing treatments were implemented (June 2015 biomass *p*‐values>0.4). Fall grazing reduced standing biomass by 30%–40% (Figure 3a, *F*_2,8_ = 7.37, *p* = 0.02). Following fall grazing, across grazing treatments, plots seeded with forage kochia and grasses had 91% and 30% more standing biomass than unseeded control plots, respectively, and unseeded control plots had 4.5 times more biomass than herbicide plots (Supporting Information Table S2; *F*_3, 120_ = 30.5, *p* < 0.0001). Planted seedlings were too small to contribute to biomass, so increased standing biomass in seeded plots relative to unseeded controls was a result of invasive species responses to the mechanical disturbance of the drill.

The spring grazing treatment reduced standing biomass by 50% relative to ungrazed plots in May 2016 (Figure 3a; *F*_2, 105_ = 17, *p* < 0.0001). Effects of fall grazing (Oct/Nov 2015) on biomass persisted into the subsequent spring for native grass plots (Figure 3b). Similar patterns were apparent for control and kochia plots, whereas herbicide plots maintained low biomass across all three grazing treatments (Figure 3b; grazing × plot type *F*_2, 119_ = 7.7, *p* < 0.0001).

Across seeded plots and controls, fall standing biomass was 23% *B. tectorum*, 74% forbs (mostly nonnative annuals), and 3% resident native grasses (mostly *P. secunda*) by weight (Supporting Information Table S2). Spring biomass averaged across controls and seeded plots included 60% *B. tectorum*, 22% forbs (mostly nonnative annuals), 17% resident native grasses (mostly *P. secunda*), and 0.5% seeded species (Supporting Information Table S2). Grazing treatments and interactions among grazing treatments and plot types had no significant effects on litter cover, invasive species cover, or invasive species density in 2015 or 2016 (all *p*‐values > 0.05; Supporting Information Table S3).

### 3.2 Grazing treatments reduced seedling densities

**Figure 4:**
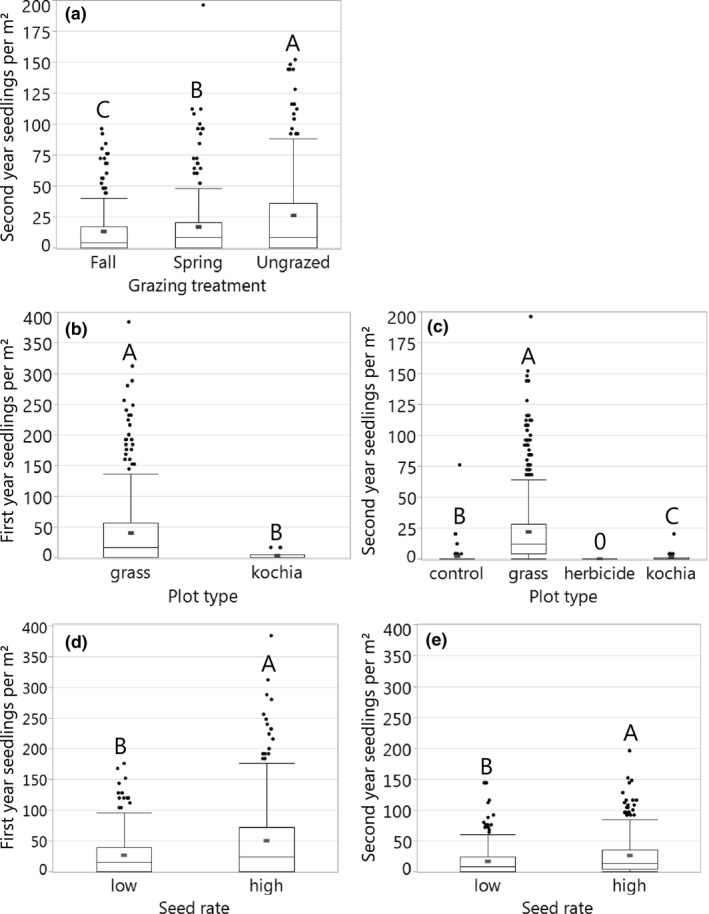
Overall effects of (a) grazing treatments in year 2, plot types in (b) year 1 and (c) year 2, and seed rate treatments in (d) year 1 and (e) year 2 on seeded species densities. Red bars are means, and box plots display variability. Within each panel, treatments sharing capital letters did not differ significantly in seedling density (Tukey HSD). In (c), all herbicide plots had zero seedlings

The grazing × establishment model revealed that when compared to densities in ungrazed paddocks, second‐year grass and kochia seedling densities were 50% lower in fall‐grazed and 36% lower in spring‐grazed paddocks (Figure 4a, *F*_2,4_ = 7.08, *p* = 0.049). Plots seeded with native grasses had almost 12 times more first‐year seedlings of planted species than forage kochia plots (Figure 4b, *F*_1,69_ = 25.7, *p* < 0.0001) and nine times more second‐year seedlings than either unseeded controls or forage kochia plots (Figure 4c, *F*_2, 121_ = 31.9, *p* < 0.0001).

### 3.3 Restoration treatments muted negative effects of grazing on seeded grasses

The planting strategies model indicated that across grazing treatments, the higher seed rate yielded approximately twice as many first‐year grass seedlings and 1.5 times as many second‐year seedlings as the lower seed rate (Figure 4d,e; Year 1: *F*_1,60_ = 17.6, *p* < 0.0001; Year 2: *F*_1,53_ = 14.9, *p* = 0.0003). Between year one and year two, seedling densities declined by 36% in the low and 50% in the high seed rate plots.

**Figure 5:**
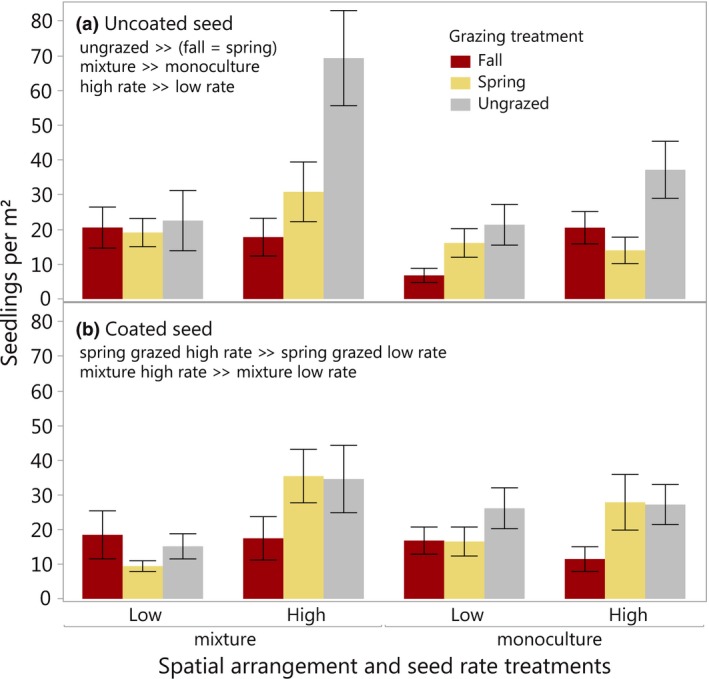
Seedling densities by grazing treatments, seed rates and spatial arrangements in (a) uncoated and (b) coated subplots. In uncoated subplots, densities were affected by grazing treatments, seed rates, and spatial planting arrangements (all main effects significant). In coated subplots, differences among treatments were more muted (grazing × seed rate and spatial arrangement × seed rate significant)

The planting strategies model also indicated a three‐way interaction among seed rate, grazing, and seed coating treatments (*F*_2,58_ = 4.84, *p* = 0.01) for second‐year seedlings. To better understand this interaction, separate models were examined for coated and uncoated seed treatments. Uncoated grass seeds in ungrazed plots produced more seedlings than uncoated seeds in either fall‐ or spring‐grazed plots, whereas fall‐ and spring‐grazed plots had similar seedling densities (Figure 5a; *F*_2,4_ = 8.39, *p* = 0.04). Across grazing treatments, seedlings from uncoated seeds were more abundant in mixtures than monocultures (*F*_1,52_ = 4.63, *p* = 0.04), and more abundant in higher than lower seed rate plots (Figure 5a; *F*_1,52_ = 11.81, *p* = 0.001). Grazing effects were not influenced by spatial planting strategy or seed rate (all interaction *p*‐values >0.05).

For coated seeds, differences among grazing treatments were more muted. Among grazing treatments, the highest seedling densities were found in spring‐grazed plots planted at high rates, which had significantly more seedlings than spring‐grazed plots planted at low rates (Figure 5b; Grazing × Seed rate *F*_2,53_ = 4.60, *p* = 0.01). All other grazing and seed rate combinations produced intermediate seedling densities. For coated seeds, mixture plots planted at high rates had more seedlings than mixture plots planted at low rates, while monoculture plots had intermediate seedling densities (Figure 5b; Spatial arrangement × Seed rate *F*_1,53_ = 4.31, *p* = 0.04).

### 3.4 Species that produced the most seedlings were also the most sensitive to grazing

**Figure 6:**
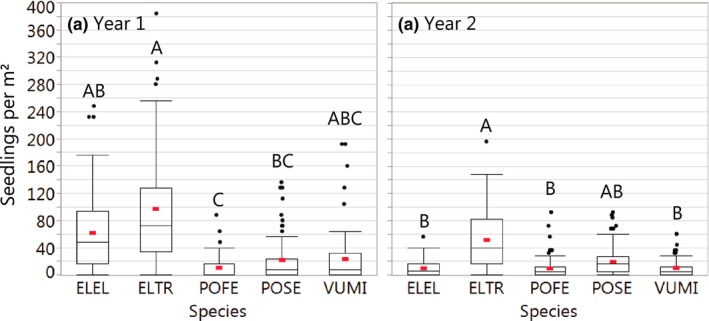
Densities of (a) first‐year and (b) second‐year grass seedlings by species for monoculture strip plots. Red bars are means. Within each panel, densities of species sharing capital letters did not differ significantly (Tukey HSD). ELEL: *Elymus* *elymoides*; ELTR: *E* *. trachycaulus*; POFE: *Poa* *fendleriana*; POSE: *P* *. secunda*; and VUMI: *Vulpia* *microstachys*

The grass species model showed that in year one, averaged across seed rates, *E. trachycaulus* produced four to nine times as many seedlings as either *Poa* species, and *E. elymoides* produced almost five times as many seedlings as *P. fendleriana* (Figure 6a, *F*_4, 270_ = 33.0, *p* < 0.0001). Between years one and two, *Poa* seedling densities did not change appreciably, but densities of *E. trachycaulus* and *V. microstachyus* declined by roughly 50%, and densities of *E. elymoides* declined by >80% (Figure 6b). In year two, *E. trachycaulus* had at least five times as many seedlings as *E. elymoides*, *V. microstachyus*, or *P. fendleriana*, while *P. secunda* had intermediate seedling densities (Figure 6b, *F*_4, 247_ = 34.5, *p* < 0.0001). In year two, we also observed two three‐way interactions in the grass species model; one among species, coating treatments, and grazing treatments (*F*_8, 247_ = 2.79, *p* = 0.006), and one among seed rates, coating treatments, and grazing treatments (*F*_2,30_ = 5.19, *p* = 0.01). Individual models were constructed for each species to better understand these interactions.

**Figure 7:**
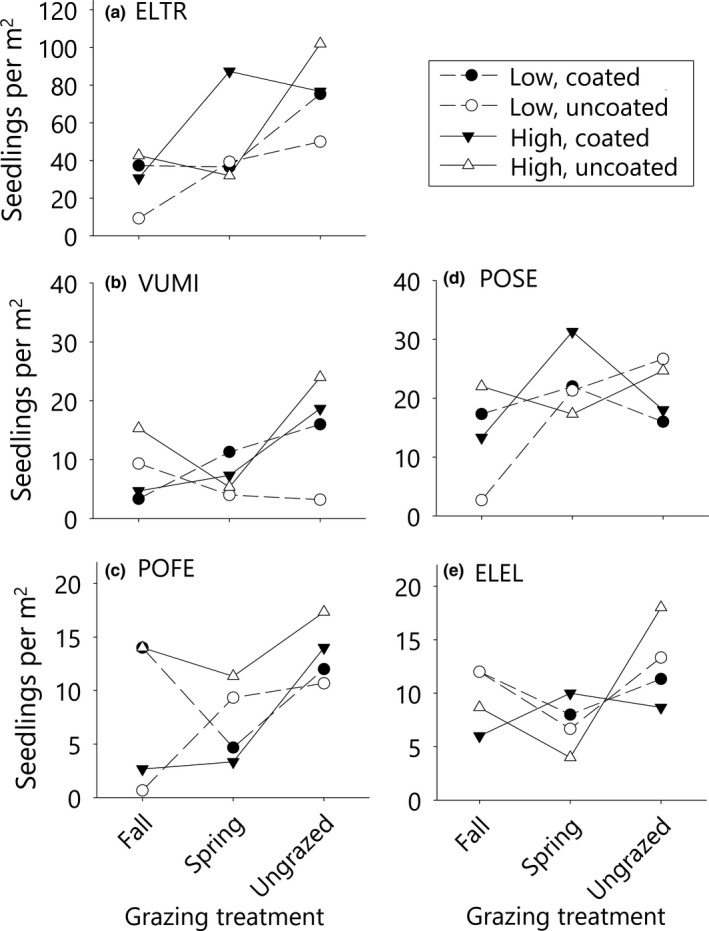
Effects of grazing, seed rate and seed coating treatments on species planted in monoculture strip plots. Three‐way interactions among grazing, seed coating, and seed rate treatments were significant for *Elymus trachycaulus*, *Poa secunda*, and *Vulpia microstachys *and marginal (*p* = 0.08) for *P. fendleriana*. See Figure 6 for acronym definitions

For all species except *E. elymoides*, there was some evidence for a three‐way interaction among seed rate, seed coating, and grazing treatments (*E. trachycaulus* *F*_2,30_ = 4.15, *p* = 0.03, *V. microstachys* *F*_2,29_ = 3.32, *p* = 0.05, *P. secunda* *F*_2,30_ = 3.22, *p* = 0.05, *P. fendleriana* *F*_2,30_ = 2.78, *p* = 0.08). For three species (*E. trachycaulus*, *P. secunda*, and *P. fendleriana*), the interaction was driven by poor performance of the low seed rate, uncoated, fall‐grazed treatment combination (Figure 7). For all four species, some combination of coating, higher seed rate, or deferred grazing (spring‐grazing or no‐grazing) led to higher second‐year seedling densities, but effect strengths varied by species (Figure 7). For *E. trachycaulus*, the highest seedling densities were found in ungrazed or spring‐grazed plots planted at the high rate with coated seed. For *P. fendleriana*, most treatments had similar seedling densities, but densities were markedly lower for uncoated seed planted in fall‐grazed, low rate plots, and coated seed planted in high rate, spring‐ or fall‐grazed plots. *Poa secunda* had relatively high seedling densities in all treatment combinations other than the fall‐grazed, uncoated, low rate combination. Results for *V. microstachys*, the only annual species, were slightly different. Coated seeds produced higher seedling densities in ungrazed plots than in fall‐grazed plots, while uncoated seeds had inconsistent responses to grazing. Across coating treatments, ungrazed, high rate plots had the highest second‐year seedling densities for *V. microstachys*. For all five species, average seedling densities did not differ significantly between spring‐grazed and ungrazed plots (Figure 7, Supporting Information Table S4).

## 4 DISCUSSION

Interrupting the invasive grass‐wildfire cycle in dryland ecosystems is a challenge worldwide, and is especially pressing in areas like the Great Basin where ecosystem conversion has occurred at broad scales (DiTomaso, 2000; Mack, 1981). We hypothesized that it might be possible to combine targeted grazing with high‐input, spatially strategic restoration to create patches of self‐sustaining, fire‐resistant vegetation. The creation of such patches is key for the long‐term recovery of desired ecosystem functions in highly invaded systems. In the first two years of this long‐term study, we found that targeted grazing during the fall or spring of the second growing season reduced standing biomass but also reduced densities of species planted in experimental greenstrips. Density reductions were mitigated by increasing seeding rates, delaying grazing, and selecting grazing‐tolerant species. Understanding how initial restoration approaches and repeated grazing treatments influence future densities of adult plants at this site requires longer‐term observations, and future assessments are planned. However, because mortality risk declines substantially after the first or second growing season (Leger & Goergen, 2017), second‐year results can provide important insights about how to address seedling establishment, which has been identified as a critical restoration bottleneck in dryland ecosystems (James et al., 2011).

### 4.1 Tradeoff between reducing standing biomass and seedling production

The flammability of annual grasses has increased both the frequency and intensity of fire in invaded dryland systems (Alba et al., 2015; Balch et al., 2013; D'Antonio & Vitousek, 1992). Our experiment revealed a clear tradeoff between reducing fuel loads and restoring desirable grasses in invaded sagebrush steppe. When results were averaged across all planting treatments, ungrazed paddocks had the highest second‐year seedling densities, but also the highest standing biomass and therefore the greatest fuel loads. The relative importance of these two factors for the long‐term persistence of seeded species and their ability to reduce wildfire spread remains unclear. For example, early reductions in seedling density would not necessarily result in lower densities of adult plants if competition among planted seedlings is strong (Mangla, Sheley, James, & Radosevich, 2011), and high seedling densities will not reduce fire risk in sites that burn before plants reach adulthood. Contrary to our prediction, our data suggest that targeted spring‐grazing (deferred until the second year) in sites recently seeded with native grasses may provide more balance between the dual objectives of seedling establishment and wildfire protection than targeted fall grazing. Spring grazing reduced standing biomass by 50% but seedling densities by only 36%. In contrast, light‐to‐moderate fall grazing at the start of the second growing season reduced seedling densities by 50% and biomass by only 30%–40%. The five‐month deferment between fall and spring grazing may help to explain the milder impact of spring grazing; it is possible that older seedlings were more tolerant of defoliation, or that new green growth of surrounding vegetation in spring altered grazing preferences away from seedlings. Additionally, wet weather in spring 2016 likely contributed to seedling survival after spring defoliation. Across both grazing treatments, second‐year seeding densities remained relatively high (>10 per m^2^ across most treatments). Based on results from our ungrazed plots, it seems likely that a longer (>1 year) deferment period would lead to higher seedling densities, though such a treatment would also likely increase fuel loads. Overall, while we observed the expected direct negative effects on seedlings, targeted grazing treatments also had the expected positive effects on fuel characteristics, and could potentially improve restoration outcomes over the long‐term by reducing competition from invasive annuals and other nonseeded species (see indirect pathways in Figure 1).

When compared to native greenstrip plots, plots seeded with kochia had similar biomass but fewer seedlings, suggesting that during the establishment phase, seeding with native grasses can produce similar or even better results in terms of seedlings grown per unit of fuel load. Herbicide was extremely successful at reducing fuel loads, but provided no other ecosystem services (e.g., forage or biodiversity benefits). Without continued maintenance, it seems likely that these brownstrips will again become dominated by invasive species (Merriam et al., 2006).

### 4.2 Strategic restoration to mitigate tradeoffs

In experimental greenstrip plots, our analysis of planting strategies revealed that several strategies improved seedling establishment and/or minimized differences among grazing treatments. The most dramatic improvements in seedling density were achieved by doubling the seeding rate, which aligns well with previous studies emphasizing the importance of propagule pressure as a driver of restoration outcomes (Mazzola et al., 2010; Schantz, Sheley, James, & Hamerlynck, 2016; Seabloom, Harpole, Reichman, & Tilman, 2003). In this study, beneficial effects of higher seed rates persisted into the second growing season, and minimized the negative impacts of grazing treatments: seedling densities in the high rate, fall‐ or spring‐grazed plots were similar to densities in ungrazed, low rate plots (Figure 5).

Seed coating has been shown to improve plant establishment in dryland systems (Madsen, Davies, Boyd, Kerby, & Svejcar, 2016; Madsen, Zvirzdin, Roundy, & Kostka, 2014). In this study, coating did not have any significant main effects on seedling establishment. Rather, it interacted in complex ways with other treatments and may have muted differences among grazing treatments. For uncoated seeds, ungrazed plots produced substantially more seedlings than either fall‐ or spring‐grazed plots (Figure 5). For coated seeds, the highest seedling densities were found in spring‐grazed and ungrazed plots planted at high rates, while most other grazing and seed rate combinations produced similar seedling densities (Figure 5). Overall, grazing treatments had a much weaker negative effect on seedling densities in subplots planted with coated seeds. The surfactant coating that was applied to the seeds has been shown in previous studies to improve seedling survival under drought stress (Ahmed et al., 2018; Madsen et al., 2012, 2014 ; Madsen, Kostka, et al., 2016). We hypothesize that in this dryland study the soil surfactant in the coating helped produce more robust plants that were able to recover quicker after the grazing treatment. However, in ungrazed, high rate plots, uncoated seedling densities were higher than coated seedling densities (Figure 5). We hypothesize that in these ungrazed plots, coated seeds were mature enough to begin self‐thinning processes during the second growing season. The development of seed coating technologies for rangeland applications are in their infancy; this study provides justification for further research on how coating technologies can help seeded plants overcome factors limiting their success in rangeland systems.

We found that seed mixtures produced more total seedlings than monoculture applications, particularly when planted with at high rates. This pattern parallels results from studies in other systems, which have found higher total cover of seeded species in mixtures due to high cover of a few dominant species (Porensky et al., 2012). In that work, increased cover came at the expense of reduced diversity as weaker competitors were excluded. In this study, it was impossible to assess diversity in mixture plots because species identification was unreliable. However, given the results from monoculture plots (Figure 7), it is likely that second‐year seedling communities in mixture plots were dominated by the most prolific seeded species, *Elymus trachycaulus*. If this dominant species is a strong competitor, it would be expected to benefit from less intraspecific and more interspecific competition in mixture plots (Stoll & Prati, 2001; Turnbull, Coomes, Purves, & Rees, 2007). Alternatively, mixture plantings could increase seedling abundance across multiple species by reducing microsite‐scale competition for the same resources, if intraspecific competition is stronger than interspecific competition at the seedling emergence stage (Leger & Espeland, 2010). Future assessments will determine whether increased seedling densities in mixture plots occurred at the expense of species diversity.

### 4.3 Species‐level responses revealed multiple strategies

Grasses as a guild are highly grazing‐tolerant, but species differ considerably in their responses to herbivory (Adler, Milchunas, Lauenroth, Sala, & Burke, 2004). Our grass species analysis demonstrated that targeted grazing can reduce seedling establishment of several native grasses, and also revealed variation in species sensitivity to grazing treatments. For example, *Elymus trachycaulus* was very successful at seedling establishment, but seedlings showed the greatest negative response to both fall and spring grazing treatments (Figure 7). In contrast, *Poa secunda *produced relatively few seedlings in the first growing season, but was unaffected by grazing and had high survival across all treatments. By the second growing season, seedling density of *P. secunda* did not differ statistically from *E. trachycaulus *across grazing treatments (Figure 6). Among the seeded species, *E. trachycaulus* and *P. secunda* may represent two ends of a survival strategy gradient, in which species that produce fewer seedlings are also more tolerant of disturbances such as grazing (Briske, 1996). In the case of small‐statured *P. secunda*, grazing avoidance might be a key element of seedling success.

It is important to note that our grazing treatments were generally different than those most rangelands experience. Targeted grazing is intended as a vegetation management tool, not as a typical grazing system. Although paddocks were ~20 ha in size, larger rangeland areas typically offer greater dietary and behavioral choices for grazing animals. Though concentration of animals into smaller paddocks is an effective technique for reducing biomass in specific areas, it can also limit diet preference choices and stimulate more uniform acquisition behavior. Thus, our results are most relevant to projects that plan to use targeted grazing in smaller areas, such as for fuels reduction or firebreak establishment. The appropriate numbers and timing of targeted grazing doses will vary considerably with precipitation amounts, seasonality, and periodicity over time and space. For managers designing grazing dose applications and setting postgrazing residual biomass targets, we recommend considering the local availability of grazing animals, potential biomass production given recent and forecasted precipitation, the phenology and palatability of targeted species, and the phenology and palatability of co‐occurring desirable species.

## 5 CONCLUSIONS

Innovative management and restoration approaches are needed to prevent the continued loss of desired ecosystem services in sagebrush steppe, one of North America's most threatened ecosystems (Davies et al., 2011). In systems threatened by invasive plants and increased wildfire frequency, livestock can provide opportunities for targeted vegetation management, and restoration treatments can affect seedling responses to grazing. Understanding how nonnative plants and animals interact can facilitate the use of herbivores as management tools to influence plant community composition in ways that favor desirable species and reduce problematic ones (Davies et al., 2009; Marty, 2005). We explored interactions between targeted grazing and seedling establishment, which has been identified as a critical bottleneck for restoration in dryland areas where annual invasive grasses are highly competitive with more desirable species (James et al., 2011). Though future assessments are needed to clarify long‐term outcomes, our study identified clear tradeoffs between establishing native plants and using livestock to reduce the spread of wildfires, and also demonstrated that potentially undesirable tradeoffs can be minimized by boosting seed rates or using grazing‐tolerant species. Our results indicate that similar efforts to use targeted grazing as a restoration tool in other dryland systems should incorporate a range of species in initial trials, and consider seeding at higher rates when grazing animals are introduced during seedling establishment.

## CONFLICT OF INTEREST

None declared.

## AUTHOR CONTRIBUTIONS

Conceived and designed experiment: LMP, EAL, MAW, BLP, MDM. Performed experiment: LMP, EAL, MDM, BLP, MAW. Analyzed data: LMP. Interpreted results and drafted manuscript: LMP, EAL, MAW. Revised manuscript and approved submission: LMP, EAL, MDM, BLP, MAW.

## DATA ACCESSIBILITY

Data on seedling densities, biomass and cover will be stored on Dryad before publication.
