# Supplementary material for: Combining active restoration and targeted grazing to establish native plants and reduce fuel loads in invaded ecosystems
Source: Ecol Evol. 2018 Dec 11;8(24):12533–46. doi: 10.1002/ece3.4642 (PMC6309004; doi:10.1002/ece3.4642)
Supplement: Supplementary file 1 [file ECE3-8-12533-s001.docx]

**Supporting Information**

**Table S1.** Estimated monthly precipitation for the years of the study, from PRISM Climate Group. Soil series present at the site include Tomera-Cherry Spring Association (60.5%), Whirlo gravelly silt loam, 2-8% slopes (12.6%), Orovada gravelly fine sandy loam (10.1%), McConnel-Blackhawk complex (8.8%), and Cherry Spring-Cortez-Chiara association (8%).

| **Month** | **Precipitation (inches) averaged across four PRISM grid cells intersecting the site** | **Project activities** |
| --- | --- | --- |
| 2014-01 | 0.5225 |  |
| 2014-02 | 1.6875 |  |
| 2014-03 | 1.4275 |  |
| 2014-04 | 0.9175 |  |
| 2014-05 | 1.0425 |  |
| 2014-06 | 0.305 | Baseline data collection |
| 2014-07 | 0.435 |  |
| 2014-08 | 1.005 |  |
| 2014-09 | 0.535 |  |
| 2014-10 | 0.0275 | Planting |
| 2014-11 | 1.245 |  |
| 2014-12 | 1.4375 |  |
| 2015-01 | 0.37 |  |
| 2015-02 | 0.245 |  |
| 2015-03 | 0.2325 |  |
| 2015-04 | 0.8475 |  |
| 2015-05 | 2.4125 | Year one data collection |
| 2015-06 | 0.68 |  |
| 2015-07 | 1.6125 |  |
| 2015-08 | 0.385 |  |
| 2015-09 | 0.2875 |  |
| 2015-10 | 1.43 | Fall grazing |
| 2015-11 | 1.76 |  |
| 2015-12 | 2.055 |  |
| 2016-01 | 1.8425 |  |
| 2016-02 | 0.2075 |  |
| 2016-03 | 1.3475 |  |
| 2016-04 | 2.0075 | Spring grazing |
| 2016-05 | 0.99 | Year two data collection |
| 2016-06 | 0.1425 |  |
| 2016-07 | 0.01 |  |
| 2016-08 | 0.045 |  |
| 2016-09 | 0.865 |  |
| 2016-10 | 2.3225 |  |
| 2016-11 | 0.4825 |  |
| 2016-12 | 2.1675 |  |

**Table S2.** Standing biomass data (mean  ± 1SE, g / m^2^) by sampling date, plot type and functional group.

| **Nov-15** |  |  |  |  |  |
| --- | --- | --- | --- | --- | --- |
| **Plot Type** | **Grazing Treatment** | **Weight (g), Cheatgrass** | **Weight (g), Forb** | **Weight (g), Native Grass** |  |
| Control | fall | 4.23 ± 1.19 | 19.49 ± 13.21 | 0.00 ± 0.00 |  |
| Control | spring | 9.84 ± 2.50 | 19.41 ± 7.12 | 1.02 ± 0.56 |  |
| Control | ungrazed | 8.22 ± 1.54 | 26.62 ± 10.30 | 2.28 ± 1.07 |  |
| Grass | fall | 5.38 ± 0.94 | 24.59 ± 5.35 | 0.01 ± 0.01 |  |
| Grass | spring | 11.60 ± 1.61 | 28.69 ± 5.46 | 0.65 ± 0.38 |  |
| Grass | ungrazed | 10.50 ± 2.00 | 35.59 ± 6.87 | 1.72 ± 0.71 |  |
| Kochia | fall | 6.30 ± 0.87 | 32.26 ± 13.99 | 0.00 ± 0.00 |  |
| Kochia | spring | 11.53 ± 3.51 | 39.93 ± 13.95 | 7.23 ± 4.05 |  |
| Kochia | ungrazed | 22.54 ± 1.15 | 54.25 ± 27.01 | 0.00 ± 0.00 |  |
| Herbicide | fall | 1.39 ± 0.52 | 0.25 ± 0.16 | 0.00 ± 0.00 |  |
| Herbicide | spring | 2.51 ± 1.18 | 8.76 ± 8.76 | 0.23 ± 0.22 |  |
| Herbicide | ungrazed | 5.60 ± 2.62 | 0.00 ± 0.00 | 1.74 ± 1.42 |  |
|  |  |  |  |  |  |
| **Jun-16** |  |  |  |  |  |
| **Plot Type** | **Grazing Treatment** | **Weight (g), Cheatgrass** | **Weight (g), Forb** | **Weight (g), Native Grass** | **Weight (g), Seedlings of planted species** |
| Control | Fall | 51.39 ± 10.01 | 16.94 ± 3.67 | 5.21 ± 3.48 | 0.00 ± 0.00 |
| Control | Spring | 62.44 ± 6.00 | 10.15 ± 3.46 | 3.13 ± 1.61 | 0.01 ± 0.01 |
| Control | Ungrazed | 78.14 ± 17.13 | 42.93 ± 9.49 | 21.70 ± 10.21 | 0.00 ± 0.00 |
| Grass | Fall | 70.97 ± 8.35 | 26.69 ± 5.46 | 1.01 ± 0.61 | 0.38 ± 0.08 |
| Grass | Spring | 65.78 ± 8.65 | 20.27 ± 3.66 | 5.29 ± 2.58 | 0.38 ± 0.04 |
| Grass | Ungrazed | 92.38 ± 13.15 | 40.92 ± 5.34 | 11.51 ± 3.99 | 0.29 ± 0.11 |
| Kochia | Fall | 54.73 ± 22.98 | 18.88 ± 8.22 | 14.97 ± 14.97 | 1.77 ± 1.12 |
| Kochia | Spring | 85.87 ± 16.21 | 13.49 ± 4.20 | 0.00 ± 0.00 | 0.00 ± 0.00 |
| Kochia | Ungrazed | 185.06 ± 32.59 | 42.69 ± 14.48 | 0.00 ± 0.00 | 0.60 ± 0.60 |
| Herbicide | Fall | 0.33 ± 0.11 | 0.15 ± 0.15 | 0.30 ± 0.30 | 0.00 ± 0.00 |
| Herbicide | Spring | 0.33 ± 0.11 | 1.36 ± 1.33 | 0.20 ± 0.20 | 0.00 ± 0.00 |
| Herbicide | Ungrazed | 0.52 ± 0.34 | 0.42 ± 0.10 | 6.30 ± 5.68 | 0.00 ± 0.00 |

**Table S3.** Statistical results for analyses of litter cover, invasive species cover, and invasive species density. In least squared means tables, treatments sharing letters are not significantly different based on Tukey HSD tests.

**Fixed Effect Tests for Litter Cover 2015**

| **Source** | **Nparm** | **DF** | **DFDen** | **F Ratio** | **Prob > F** |  |
| --- | --- | --- | --- | --- | --- | --- |
| Grazing | 2 | 2 | 12.27 | 1.4175 | 0.2794 |  |
| Plot Type | 3 | 3 | 374.7 | 13.6097 | <.0001* |  |
| Grazing*Plot Type | 6 | 6 | 328.7 | 1.8037 | 0.0977 |  |

**Least Squares Means Table for Litter Cover 2015 by Plot Type**

| **Level** | **Least Sq Mean** |  | **Std Error** |
| --- | --- | --- | --- |
| Control **A** | 3.9877007 |  | 0.28088847 |
| Grass **B** | 2.6336225 |  | 0.18063764 |
| Herbicide **A** | 3.4961147 |  | 0.32940801 |
| Kochia **AB** | 3.2895082 |  | 0.34285327 |

**Fixed Effect Tests for Litter Cover 2016**

| **Source** | **Nparm** | **DF** | **DFDen** | **F Ratio** | **Prob > F** |  |
| --- | --- | --- | --- | --- | --- | --- |
| Grazing | 2 | 2 | 15.52 | 0.5866 | 0.5681 |  |
| Plot Type | 3 | 3 | 408.7 | 5.5802 | 0.0009* |  |
| Grazing* Plot Type | 6 | 6 | 403.2 | 0.5582 | 0.7635 |  |

**Least Squares Means Table for Litter Cover 2016 by Plot Type**

| **Level** | **Least Sq Mean** |  | **Std Error** |
| --- | --- | --- | --- |
| Control **A** | 1.3210857 |  | 0.14533397 |
| Grass **B** | 1.1227111 |  | 0.13442726 |
| Herbicide **A** | 1.7794739 |  | 0.25683378 |
| Kochia **AB** | 1.3930634 |  | 0.19406108 |

**Fixed Effect Tests for Invasive Species Cover 2015**

| **Source** | **Nparm** | **DF** | **DFDen** | **F Ratio** | **Prob > F** |  |
| --- | --- | --- | --- | --- | --- | --- |
| Grazing | 2 | 2 | 11.3 | 0.1302 | 0.8792 |  |
| Plot Type | 3 | 3 | 271.1 | 1.0630 | 0.3652 |  |
| Grazing*Plot Type | 6 | 6 | 260.9 | 0.9679 | 0.4474 |  |

**Fixed Effect Tests for Invasive Species Cover 2016**

| **Source** | **Nparm** | **DF** | **DFDen** | **F Ratio** | **Prob > F** |  |
| --- | --- | --- | --- | --- | --- | --- |
| Grazing | 2 | 2 | 8.108 | 0.9891 | 0.4127 |  |
| Plot Type | 3 | 3 | 223.5 | 72.3580 | <.0001* |  |
| Grazing*Plot Type | 6 | 6 | 215 | 0.7286 | 0.6270 |  |

**Least Squares Means Table for Invasive Cover 2016 by Plot Type**

| **Level** | **Least Sq Mean** |  | **Std Error** |
| --- | --- | --- | --- |
| Control **A** | 69.699074 |  | 9.530949 |
| Grass **A** | 70.195979 |  | 9.456952 |
| Herbicide **B** | 12.340278 |  | 10.042182 |
| Kochia **A** | 68.741404 |  | 10.655334 |

**Fixed Effect Tests for Invasive Species Density 2015**

| **Source** | **Nparm** | **DF** | **DFDen** | **F Ratio** | **Prob > F** |  |
| --- | --- | --- | --- | --- | --- | --- |
| Grazed | 2 | 2 | 7.593 | 0.1404 | 0.8712 |  |
| Plot Type | 3 | 3 | 133.4 | 1.9486 | 0.1248 |  |
| Grazed*Plot Type | 6 | 6 | 127.9 | 0.4407 | 0.8504 |  |

**Fixed Effect Tests for Invasive Species Density 2016**

| **Source** | **Nparm** | **DF** | **DFDen** | **F Ratio** | **Prob > F** |  |
| --- | --- | --- | --- | --- | --- | --- |
| Grazed | 2 | 2 | 6.789 | 0.4428 | 0.6595 |  |
| Fuelbreak/control | 3 | 3 | 229.8 | 67.4418 | <.0001* |  |
| Grazed*Fuelbreak/control | 6 | 6 | 231.6 | 1.5413 | 0.1655 |  |

**Least Squares Means Table for Invasive Species Density 2016 by Plot Type**

| **Level** | **Least Sq Mean** |  | **Std Error** |
| --- | --- | --- | --- |
| Control **A** | 4.9439848 |  | 0.15802529 |
| Greenstrip **A** | 4.7577586 |  | 0.15330577 |
| Herbicide **B** | 1.0771706 |  | 0.28291794 |
| Kochia **A** | 4.9815784 |  | 0.35693214 |

**Table S4.** Planted native species, seed rates, and mean  ± 1SE second-year seedling densities per m^2^ in monoculture plots for different combinations of grazing and seed rate treatments. For each species, treatments sharing letters are not significantly different.

| **Species** | **Low rate** | **High rate** | **Ungrazed**  **high** | **Ungrazed**  **low** | **Spring**  **high** | **Spring**  **low** | **Fall**  **high** | **Fall**  **low** |
| --- | --- | --- | --- | --- | --- | --- | --- | --- |
|  | PLS^*^ kg/ha | | Second growing season plants/m^2^ | | | | | |
| *Elymus elymoides* (squirreltail) | 3.05 | 6.11 | 13.3 ± 4.4^a^ | 12.3 ± 3.4^a^ | 7.0 ± 2.4^a^ | 7.3 ± 1.3^a^ | 7.3 ± 2.5^a^ | 12.0 ± 5.2^a^ |
| *Elymus trachycaulus* (slender wheatgrass) | 4.34 | 8.68 | 89.3 ± 11.2^a^ | 62.7 ± 12.2^ab^ | 59.7 ± 16.8^ab^ | 38.0 ± 9.9^abc^ | 36.7 ± 10.2^bc^ | 23.3 ± 6.3^c^ |
| *Poa fendleriana* (muttongrass) | 0.65 | 1.30 | 15.7 ± 7.9^a^ | 11.3 ± 5.2^a^ | 7.3 ± 2.9^a^ | 7.0 ± 2.6^a^ | 8.3 ± 3.6^a^ | 7.3 ± 5.9^a^ |
| *Poa secunda* (Sandberg's bluegrass) | 0.56 | 1.12 | 21.3 ± 8.0^a^ | 21.3 ± 7.9^a^ | 24.3 ± 7.4^a^ | 21.7 ± 6.5^a^ | 17.7 ± 5.8^a^ | 10.0 ± 4.0^a^ |
| *Vulpia microstachys*  (small fescue) | 0.61 | 1.22 | 21.3 ± 5.6^a^ | 10.2 ± 3.9^ab^ | 6.3 ± 1.5^ab^ | 7.7 ± 3.1^ab^ | 10.0 ± 5.0^ab^ | 6.3 ± 2.2^b^ |
| *Bassia prostrata*  (forage kochia) | 6.73 | | 4 ± 2.4 | | 0 ± 0 | | 0.5 ± 0.5 | |

* Seeding rates are Pure Live Seed.
